# Immunogenicity of a third dose with mRNA-vaccines in the ChAdOx1-S/BNT162b2 vaccination regimen against SARS-CoV-2 variants

**DOI:** 10.1016/j.isci.2024.110728

**Published:** 2024-08-14

**Authors:** Javier García-Pérez, Alberto M. Borobia, Mayte Pérez-Olmeda, Antonio Portolés, Luis Castaño, Magdalena Campins-Artí, María Jesús Bertrán, Mercedes Bermejo, José Ramón Arribas, Andrea López, Ana Ascaso-del-Rio, Eunate Arana-Arri, Inmaculada Fuentes Camps, Anna Vilella, Almudena Cascajero, María Teresa García-Morales, María Castillo de la Osa, Carla Pérez Ingidua, David Lora, Paloma Jiménez-Santana, Silvia Pino-Rosa, Agustín Gómez de la Cámara, Erick De La Torre-Tarazona, Esther Calonge, Raquel Cruces, Cristóbal Belda-Iniesta, José Alcamí, Jesús Frías, Antonio J. Carcas, Francisco Díez-Fuertes, CombiVacS Study Group, Lucía Díaz García, Elena Ramírez García, Enrique Seco Meseguer, Stefan Mark Stewart Balbàs, Alicia Marín Candón, Irene García García, Mikel Urroz Elizalde, Paula de la Rosa, Marta Sanz García, Cristina López Crespo, Vega Mauleón Martínez, Raquel de Madariaga Castell, Laura Vitón Vara, Rocío Prieto-Pérez, Emilio Vargas-Castrillón, Leonor Laredo, Ouhao Zhu-Huang, Teresa Iglesias, Natale Imaz-Ayo, Susana Meijide, Aitor García de Vicuña, Ana Santorcuato, Iraide Exposito, Sara de Benito, Alazne Bustinza, Mikel Gallego, Dolores García-Vázquez, Ana Belén de la Hoz, Gustavo Pérez-Nanclares, Josu Aurrekoetxea, Ines Urrutia, Rosa Martínez-Salazar, Janire Orcajo, Begoña Calvo, June Corcuera, Olaia Velasco, Anibal Aguayo, Xavier Martínez-Gómez, Susana Otero-Romero, Lluis Armadans, Blanca Borras-Bermejo, Oleguer Parés, Sonia Uriona, José Ángel Rodrigo Pendás, Cesar Llorente, José Santos, Laia Pinós, Lina Camacho, Judith Riera, Carla Sans, Antonia Agustí, Carmen Altadill, Carla Aguilar Blancafort, Gisela Gili Serrat, Aitana Plaza, Anna Feliu Prius, Maria Margarita Torrens, Esther Palacio, Gloria Torres, Julia Calonge, Elena Ballarin Alins, Eulàlia Pérez-Esquirol, Lourdes Vendrell Bosch, Marta Aldea, Eugènia Mellado, Ma Ángeles Marcos, Marta Tortajada, Lourdes E. Barón-Mira, Laura Granés, Sulayman Lazaar, Sara Herranz, Montserrat Malet, Sebastiana Quesada, Anna Llupià, Victoria Olivé, Antoni Trilla, Begoña Gómez, Elisenda González, Sheila Romero, Francisco Javier Gámez, Cristina Casals, Laura Burunat, Juan José Castelló, Patricia Fernández, Josep Lluís Bedini, Jordi Vila, Juan Carlos Hurtado, Isabel Jado, Giovanni Fedele, Concepción Perea, Mónica González, Isabel Grajera, María Ángeles Murillo, Pilar Balfagón, Irene Díaz-Marín, Gema González-Pardo

**Affiliations:** 1 Unidad de Inmunopatología del SIDA, Centro Nacional de Microbiología, Instituto de Salud Carlos III (ISCIII), Majadahonda, 28222 Madrid, Spain; 2 Centro de Investigación Biomédica en Red de Enfermedades Infecciosas (CIBERINFEC), Instituto de Salud Carlos III (ISCIII), Majadahonda, 28222 Madrid, Spain; 3 Servicio de Farmacología Clínica, Hospital Universitario La Paz, Departamento de Farmacología y Terapéutica, Facultad de Medicina, Universidad Autónoma de Madrid, IdiPAZ, 28046 Madrid, Spain; 4 Servicio de Medicina Interna, Departamento de Medicina, Facultad de Medicina, Hospital Universitario La Paz, IdiPAZ, Universidad Autónoma de Madrid, 28046 Madrid, Spain; 5 Laboratorio de Serología, Centro Nacional de Microbiología, Instituto de Salud Carlos III (ISCIII), Majadahonda, 28222 Madrid, Spain; 6 Servicio de Farmacología Clínica, Hospital Clínico San Carlos, IdISSC, 28040 Madrid, Spain; 7 Departamento de Farmacología y Toxicología, Facultad de Medicina, Universidad Complutense de Madrid (UCM), 28040 Madrid, Spain; 8 Cruces University Hospital, Bio-Bizkaia, UPV/EHU, CIBERDEM/CIBERER, Endo-ERN, Barakaldo, 48903 Vizcaya, Spain; 9 Servicio de Medicina Preventiva y Epidemiología, Hospital Universitari Vall d'Hebron, Universitat Autònoma de Barcelona, 08035 Barcelona, Spain; 10 Servicio de Medicina Preventiva y Epidemiología, Hospital Clínic de Barcelona, 08036 Barcelona, Spain; 11 Unidad de Soporte a la Investigación Clínica, Vall d’Hebron Institut de Recerca, Servicio de Farmacología Clínica, Hospital Universitari Vall d’Hebron, 08035 Barcelona, Spain; 12 Spanish Clinical Research Network − SCReN − ISCIII, 28029 Madrid, Spain; 13 Instituto de Investigación Sanitaria del Hospital Universitario 12 de octubre, 28041 Madrid, Spain; 14 Facultad de Estudios Estadísticos, Universidad Complutense de Madrid, 28040 Madrid, Spain; 15 Directorate Instituto de Salud Carlos III, 28029 Madrid, Spain

**Keywords:** Immunology, Immune response

## Abstract

CombiVacS study has demonstrated a strong immune response of the heterologous ChAdOx1-S/BNT162b2 vaccine combination. The primary outcomes of the study were to assess the humoral immune response against SARS-CoV-2, 28 days after a third dose of a mRNA vaccine, in subjects that received a previous prime-boost scheme with ChAdOx1-S/BNT162b2. Secondary outcomes extended the study to 3 and 6 months. The third vaccine dose of mRNA-1273 in naive participants previously vaccinated with ChAdOx1-S/BNT162b2 regimen reached higher neutralizing antibodies titers against the variants of concern Delta and BA.1 lineage of Omicron compared with those receiving a third dose of BNT162b2 at day 28. These differences between BNT162b2 and mRNA-1273 arms were observed against the ancestral variant G614 at day 90. Suboptimal neutralizing response was observed against BQ.1.1, XBB.1.5/XBB.1.9, and JN.1 in a relevant proportion of individuals 180 days after the third dose, even after asymptomatic Omicron breakthrough infections. EudraCT (2021-001978-37); ClinicalTrials.gov (NCT04860739).

## Highlights

• Higher NT_50_ titers was observed in mRNA-1273 arm against Delta and BA.1 at day 28
• Higher NT_50_ titers was observed in mRNA-1273 arm against G614 variant at day 90
• Suboptimal neutralization against recent Omicron variants was observed at day 180
• Asymptomatic breakthrough infections do not ensure XBB.1.5 and JN.1 neutralization

## Graphical abstract

**Figure:**
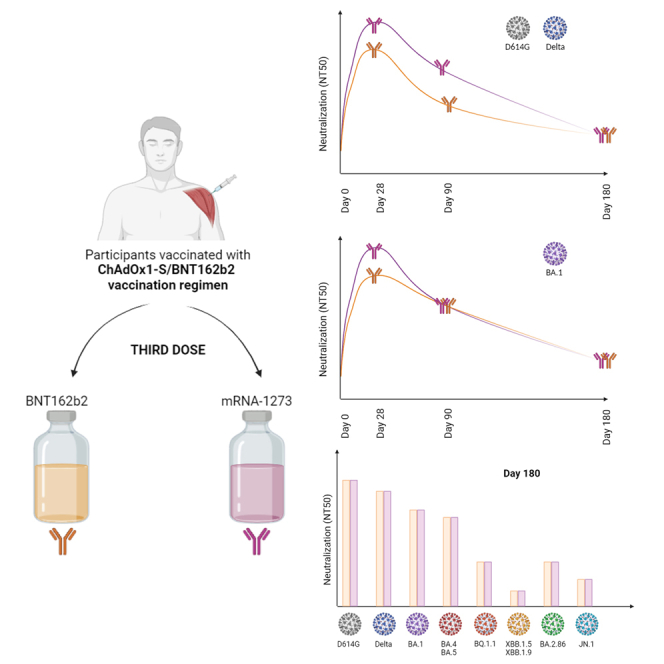

## Introduction

At the end of January 2021, the European Medicines Agency had only approved three vaccines, including two mRNA-based vaccines: BNT162b2 (Comirnaty, Pfizer-BioNTech) and mRNA-1273 (SpikeVax, Moderna-NIAID), and the adenovirus-based ChAdOx1-S vaccine (Vaxzevria, Oxford-AstraZeneca). Some cases of venous thrombosis with thrombocytopenia were firstly reported in Norway and Denmark within a month after one dose of ChAdOx1-S.^1^ Further analysis found an increased risk of cerebral venous thrombosis and other thrombosis events preferentially in women under 40 years olds after one dose of ChAdOx1-S, at very low rates of 16.1 and 36.3 per million doses, respectively.^2^ This fact, along with supply shortages, urged European countries, among others, to modify their national vaccination strategies and to explore heterologous regimens for people primed with ChAdOx1-S, being the combination with BNT162b2 the first heterologous scheme studied.^3^ A robust immune response was observed for the participants of this study after 14 and 28 days of BNT162b2 boost, detecting an antibody waning from day 14 to day 180.^3^^,^^4^ A 9.6-fold reduction in neutralizing antibody titers was observed from day 14 to day 180 against the D614G SARS-CoV-2 variant, with a relevant proportion of participants (76%) still exhibiting high neutralizing activity.^4^ However, this proportion drops to 14.3% against the Omicron variant BA.1 at day 180 of the study.^4^

Since early 2022, Omicron variant has dominated the SARS-CoV-2 pandemic.^5^ In the majority of the European countries, the initial Omicron BA.1 was later displaced by BA.2, which has further evolved to other sublineages that sequentially become dominant variants, including BA.5, BQ.1.1, XBB.1.5, and JN.1. These newly emerged SARS-CoV-2 Omicron sublineages may affect vaccine effectiveness as demonstrates its expansion in highly immunized populations.^5^^,^^6^^,^^7^^,^^8^ Different studies warning about the suboptimal neutralizing response generated by vaccination and BA.1 or BA.2 breakthrough infection in individuals vaccinated three or four times with mRNA vaccines.^9^^,^^10^^,^^11^^,^^12^^,^^13^ The loss of activity of therapeutic monoclonal neutralizing antibodies against BQ.1.1 and XBB.1.5 reinforces the idea of antibody evasion of these rising Omicron variants.^12^^,^^13^

The use of vaccines adapted for BA.5 from September 2022 does not seem to have solved the problem of the relative resistance of Omicron to the humoral response elicited by vaccination, since, despite producing high neutralizing antibodies against BA.4/BA.5, the bivalent vaccine do not produce a potent neutralizing response against the expanding variants BQ.1.1 and XBB.1 lineages.^10^ Interestingly, a recent study found that non-Omicron breakthrough infections elicited a robust cross-neutralizing activity against Omicron variants, being the time from vaccination to infection the most strongly determinant of BA.5 neutralizing capacity.^14^

Regarding heterologous vaccine schemes, a recent study has compared the ChAdOx1-S/BNT162b2 and BNT162b2/BNT162b2 regimens after a third BNT162b2 dose, showing neutralizing activity against BA.1 in 11.3% and 38.5% of participants at 6 months after the third dose, respectively. Both regimens were associated to a slower waning of SARS-CoV-2 specific IgG and neutralizing response after a third dose compared with the decrease observed after the second dose,^15^ which is in accordance with another work studying a BNT162b2 homologous vaccine scheme.^16^ Sim et al. (2023) suggest that people following the heterologous regimen (ChAdOx1-S/BNT162b2/BNT162b2) may be more susceptible to Omicron breakthrough infections but elicit a stronger cellular response than the people with three doses of BNT162b2.^15^ However, there is little information in the literature on the neutralization capacity of BQ.1.1, XBB.1.5, XBB.1.9, and BA.2.86-derived lineages for heterologous vaccine regimens.

The present study addresses humoral immune response dynamics in people primed and boosted with a ChAdOx1-S/BNT162b2 regimen after a third dose with either BNT162b2 or mRNA-1273. The primary outcomes were to assess the humoral immune response against SARS-CoV-2, 28 days after a third dose of a mRNA vaccine, in subjects that received a previous prime-boost scheme with ChAdOx1-S/BNT162b2. The secondary outcomes include to assess the humoral immune response against SARS-CoV-2, 28 days, 3 and 6 months after a third dose of a mRNA vaccine, in subjects that received a previous prime-boost scheme with ChAdOx1-S/BNT162b2. Neutralizing capacity against Delta, BA.1 and the newly emerged Omicron variants BA.4/BA.5, BQ.1.1, XBB.1.5/XBB.1.9, BA.2.86, and JN.1 is also studied at 6 months after the third dose, providing valuable data on the implication of this heterologous vaccination regimens in the capacity to neutralize the Omicron variants that have dominated the pandemic in 2023–2024.

## Results

### Study participants

**Figure 1:**
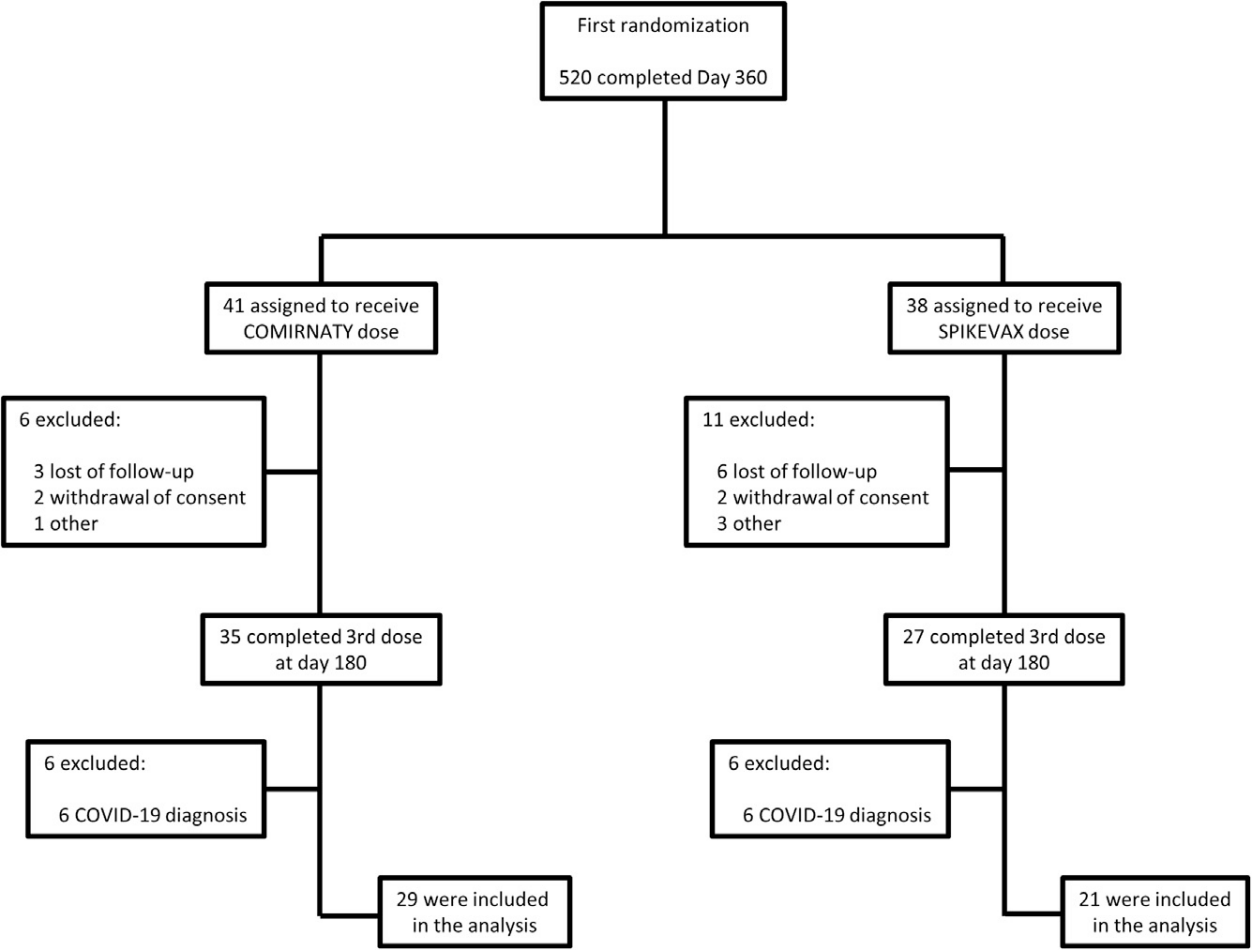
Flow chart showing the distribution of the patients included in the study

Between March 22 and April 18, 2022, 79 participants previously receiving a prime-boost with ChAdOx1-S/BNT162b2 were enrolled to receive a third dose with either BNT162b2 or mRNA-1273. At the end of the study, 50 participants completed the planned visits at days 28, 90, and 180, 29 in BNT162b2 arm and 21 in mRNA-1273 arm (Figure 1). A total of 13 (44.8%) participants were woman in BNT162b2 arm and 12 (57.1%) in mRNA-1273 arm. Half of participants were ages 18–49 years old and the mean age of participants was 48.8 (SD 7.9) (Table 1). Recent asymptomatic SARS-CoV-2 infections were assayed throughout the study, detecting IgG to nucleocapsid (N+) in 62.1% of participants vaccinated with BNT162b2, and in 52.4% of participants vaccinated with mRNA-1273 at day 180 (Table 2).

**Table 1:** Age and sex of the participants included in the study according to the vaccine administered as third dose and SARS-CoV-2 nucleocapsid IgG test results

		Age		*p* value^a^	Sex		*p* value^a^
		18–49	50–59		Female	Male	
**NC**-							

	BNT162b2	6 (54.5%)	5 (45.5%)		6 (54.5%)	5 (45.5%)	
	mRNA-1273	3 (30%)	7 (70%)	0.387	4 (40%)	6 (60%)	0.670

**NC+**							

	BNT162b2	8 (44.4%)	10 (55.6%)		7 (38.9%)	11 (61.1%)	
	mRNA-1273	8 (72.7%)	3 (27.3%)	0.249	8 (72.7%)	3 (27.3%)	0.128

a According to Fisher's exact test and comparing BNT162b2 and mRNA-1273 arms.

**Table 2:** Positivity rate of IgG to nucleocapsid in both arms throughout the study

	BNT162b2	mRNA-1273	*p* value
Baseline	8 (19.5%)	6 (17.6%)	1.0
Day 28	10 (25.0%)	7 (21.2%)	0.918
Day 90	13 (35.1%)	9 (29.0%)	0.783
Day 180	18 (62.1%)	11 (52.4%)	0.693

### Total immunoglobulins to SARS-CoV-2 spike

**Figure 2:**
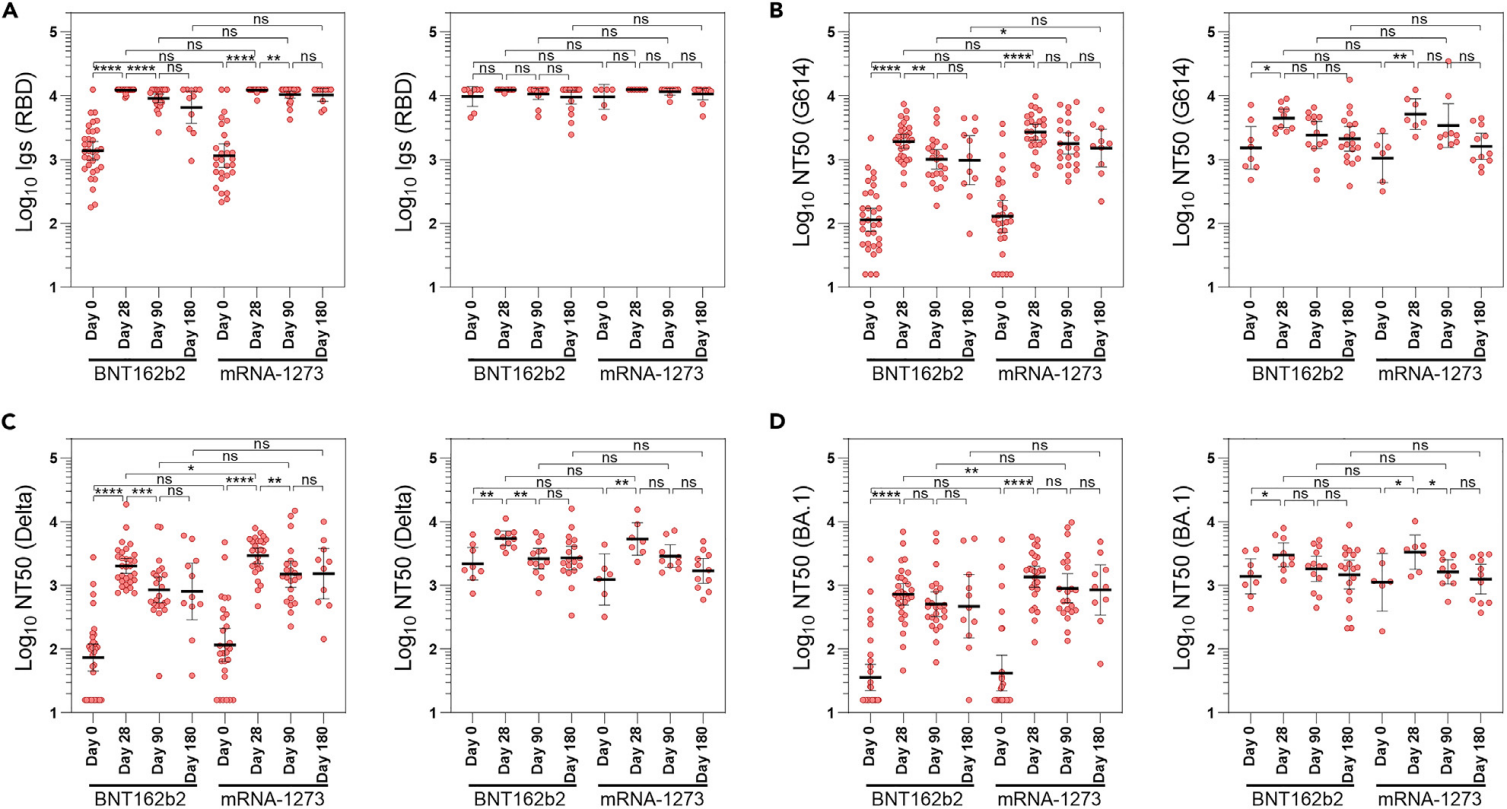
Humoral immune response against SARS-CoV-2 spike after a third vaccine dose (A–D) Total anti-RBD immunoglobulins (A) and pseudotyped neutralization assay against the ancestral SARS-CoV-2 variant G614 (B), and variants of concern Delta (C) and BA.1 sublineage of Omicron (D). Left panels in A, B, C, and D show the results for N- subjects whereas right panels show the results for N+ participants. Levels of immunoglobulins are reported in BAU/ml and neutralizing activity in neutralizing titer 50 (NT_50_). All measures were summarized as geometric mean and 95% confidence interval. Ns: *p* > 0.05; ∗*p* ≤ 0.05; ∗∗*p* ≤ 0.01; ∗∗∗*p* ≤ 0.001; ∗∗∗∗*p* ≤ 0.0001. See also Tables S1–S10.

Long-term immunogenicity of the heterologous ChAdOx1-S/BNT162b2 vaccine regimen was measured after 12 months from the second dose. Geometric mean titers (GMTs) of total SARS-CoV-2 RBD-Ig at baseline of the present study were 1382 BAU/mL (95% CI 990–1929) and 1155 BAU/mL (95% CI 754–1768) for seronegative individuals to nucleocapsid (N-) vaccinated with BNT162b2 or mRNA-1273, respectively, showing 82.1% and 85.1% decreases from the 7739 BAU/mL (95% CI 7371–8161) observed 14 days after the second dose. The third vaccine dose in BNT162b2 arm was measured in the present study for N- individuals and generated a 8.8-fold increase from 1382 BAU/mL (95% CI 990–1929) to 12221 BAU/mL (95% CI 11897–12553) at day 28, followed by a 25.1% decrease to 9158 BAU/mL (95% CI 7814–10733) at day 90 and a final 45.9% decrease to 6613 BAU/mL (95% CI 3687–11860) at day 180. On his part, the third vaccine dose of mRNA-1273 in naive individuals produced a 10.6-fold increase from 1155 BAU/mL (95% CI 754–1768) to 12243 BAU/mL (95% CI 11862–12637) at day 28, followed by a 14.8% decrease to 10432 BAU/mL (95% CI 9158–11882) at day 90 and a total decrease of 15.8%–10303 BAU/mL (95% CI 8157–13014) at day 180 (Figure 2A; Table S1). Regarding the waning of total Igs to SARS-CoV-2, statistically significant differences were observed after comparing the RBD-Ig levels at day 28 with day 90 in both BNT162b2 and mRNA-1273 groups (*p* < 0.001), but these differences disappeared by comparing days 90 and 180 (Figure 2A).

### Neutralization of SARS-CoV-2 variants

Waning of neutralizing antibodies was also studied after 12 months from the second vaccine dose. GMT of neutralizing antibodies against the G614 ancestral variant at baseline of the present study was 114 NT_50_ (95% CI 75–172) and 129 NT_50_ (95% CI 73–229) for N- individuals vaccinated with BNT162b2 or mRNA-1273, respectively, evidencing 93.2% and 94.0% decreases from the peak of 1906 NT_50_ (95% CI 1626–2234) observed at day 14 after the second vaccine dose in the interventional group.^3^ The third dose with BNT162b2 in N- individuals generated a 16.9-fold increase in the NT_50_ against the ancestral variant, from 114 (95% CI 75–172) at baseline to 1929 (95% CI 1500–2481) at day 28, followed by a 47.6% decrease to 1011 (95% CI 707–1444) at day 90 and a final 49.2% decrease to 980 (95% CI 406–2367) at day 180 (Figure 2B; Table S2). The third dose with mRNA-1273 generated a 20.9-fold increase in the neutralizing titer against the ancestral variant, from 129 NT_50_ (95% CI 73–229) at baseline to 2693 (95% CI 2026–3581) at day 28, followed by a 33.3% decrease to 1795 (95% CI 1224–2632) at day 90 and a final 43.5% decrease to 1522 (95% CI 772–3002) at day 180 (Figure 2B; Table S2). Higher neutralization potency of the ancestral variant was observed in mRNA-1273 arm compared with BNT162b2 arm for N- individuals at days 28 (*p* = 0.08) and 90 (*p* < 0.05).

Regarding the neutralizing potency of Delta variant, after the second vaccine dose (first boost with BNT162b2) a 86.8% drop in NT_50_ values were observed, from 717.1 (95% CI 587.3–876.1) at day 28–94.6 (95% CI 71.8–124.5) at day 180. After the third dose, NT_50_ values decreased from 2027 (95% CI 1540–2669) at day 28–812 (95% CI 292–2259) at day 180 for N- individuals vaccinated with BNT162b2 (59.9% decrease) and from 2927 (95% CI 2196–3900) at day 28–1525 (95% CI 615–3780) at day 180 for N- individuals from mRNA-1273 arm (47.9% decrease) (Figure 2C; Table S3). Higher NT_50_ values against Delta variant was observed in mRNA-1273 arm compared with BNT162b2 at day 28 (*p* < 0.05) and at day 90 (*p* = 0.07).

Concerning the neutralizing titers against BA.1 Omicron sublineage after the second dose, NT_50_ values drop from 144.8 (95% CI 116.7–179.9) at day 28–34.5 (95% CI 27.7–42.8) at day 180 (76.2% decrease). After the third dose, NT_50_ values decreased from 725 (95% CI 493–1065) at day 28–471 (95% CI 150–1476) at day 180 for N- individuals vaccinated with BNT162b2 (35.0% decrease) and from 1368 (95% CI 935–2003) at day 28–852 (95% CI 344–2110) at day 180 for N- individuals from mRNA-1273 arm (37.7% decrease) (Figure 2D; Table S4). Higher NT_50_ values against BA.1 variant was observed in mRNA-1273 arm compared with BNT162b2 at day 28 (*p* < 0.01).

### Effect of asymptomatic breakthrough infections on third dose immunogenicity

**Figure 3:**
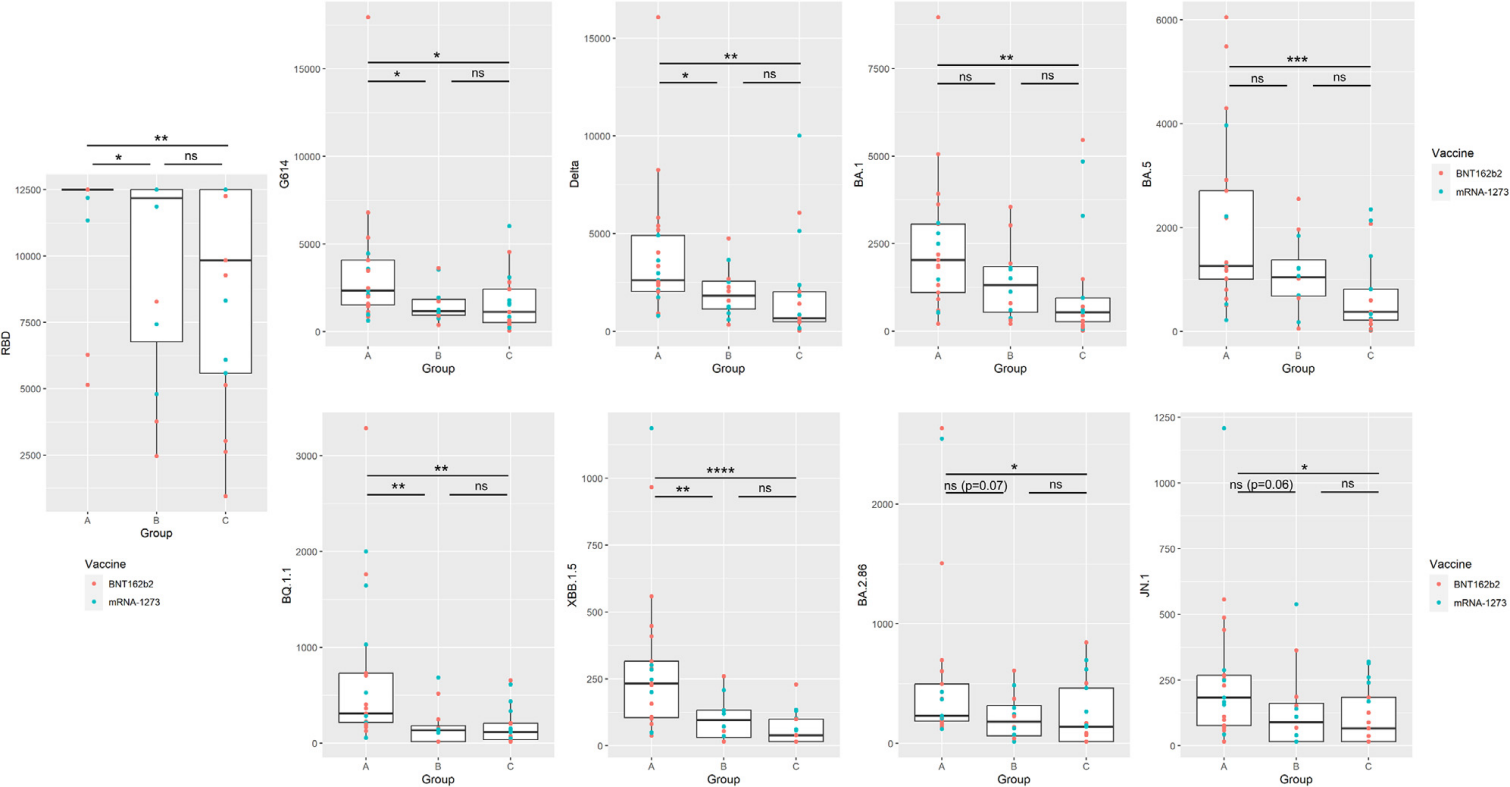
Effect of breakthrough infections in the immunogenicity of the third vaccine dose (Group A represent the results of participants with asymptomatic breakthrough infection (defined as any positive sample to SARS-CoV-2 nucleocapsid IgG during the study along with a negative sample at day 0). Group B represent participants asymptomatically infected before the third dose (defined as subjects with a positive sample to anti-nucleocapsid IgG at day 0). Group C represent participants who remained Nc- throughput the study period. Levels of immunoglobulins are reported in BAU/ml and neutralizing activity in neutralizing titer 50 (NT_50_). Data are represented as median and interquartile range. Ns: *p* > 0.05; ∗*p* ≤ 0.05; ∗∗*p* ≤ 0.01; ∗∗∗*p* ≤ 0.001; ∗∗∗∗*p* ≤ 0.0001.

In the case of the participants with a recent history of SARS-CoV-2 asymptomatic infection (N+), no statistically significant differences were found between BNT162b2 and mRNA-1273 arms at any time (Figure 2; Tables S5–S8). At baseline, 8 out of 41 (19.5%) and 6 out of 34 (17.6%) were positive to IgGs anti-N in BNT162b2 and mRNA-1273 arms, respectively. Within BNT162b2 arm, 5 participants become N+ during the first three months of the study and other 5 during the following three months. Among participants vaccinated with mRNA-1273, 3 were asymptomatically infected during the first three months and other 2 during the following three months, respectively. As expected, GMTs of total SARS-CoV-2 RBD-Ig and NT_50_ against ancestral, Delta, and Omicron BA.1 variants were higher in N+ group as compared with N- individuals at day 0 (Tables S9 and S10). Higher total anti-RBD Igs and neutralizing titers were found at day 180 in subjects with breakthrough infections compared with participants that remained N- throughout the experiment (*p* < 0.05) but also against some variants compared with people asymptomatically infected before the third dose. In the case of Omicron sublineages, these differences were especially important comparing NT_50_ values against the most evolved Omicron variants, BQ.1.1 (*p* < 0.01), XBB.1.5 (*p* < 0.01), BA.2.86 (*p* = 0.069), and JN.1 (*p* = 0.063) as opposed to the differences found against the ancestral Omicron variants BA.1 and BA.5 (*p* > 0.1) (Figure 3).

### Effect of age and sex on third dose immunogenicity

Potential age and sex differences in the immunogenicity of the third dose were also analyzed. Statistically significant differences were found in the neutralizing antibodies against all variants at day 180 after the third dose when comparing subjects under 50 years old with 50–59 years old participants and regardless of the vaccine administered (Figure S1). Regarding sex, statistically significant differences were found when comparing NT_50_ values against BA.5 and BQ.1.1 (Figure S2).

### Long-term neutralization capacity against recent Omicron lineages

**Figure 4:**
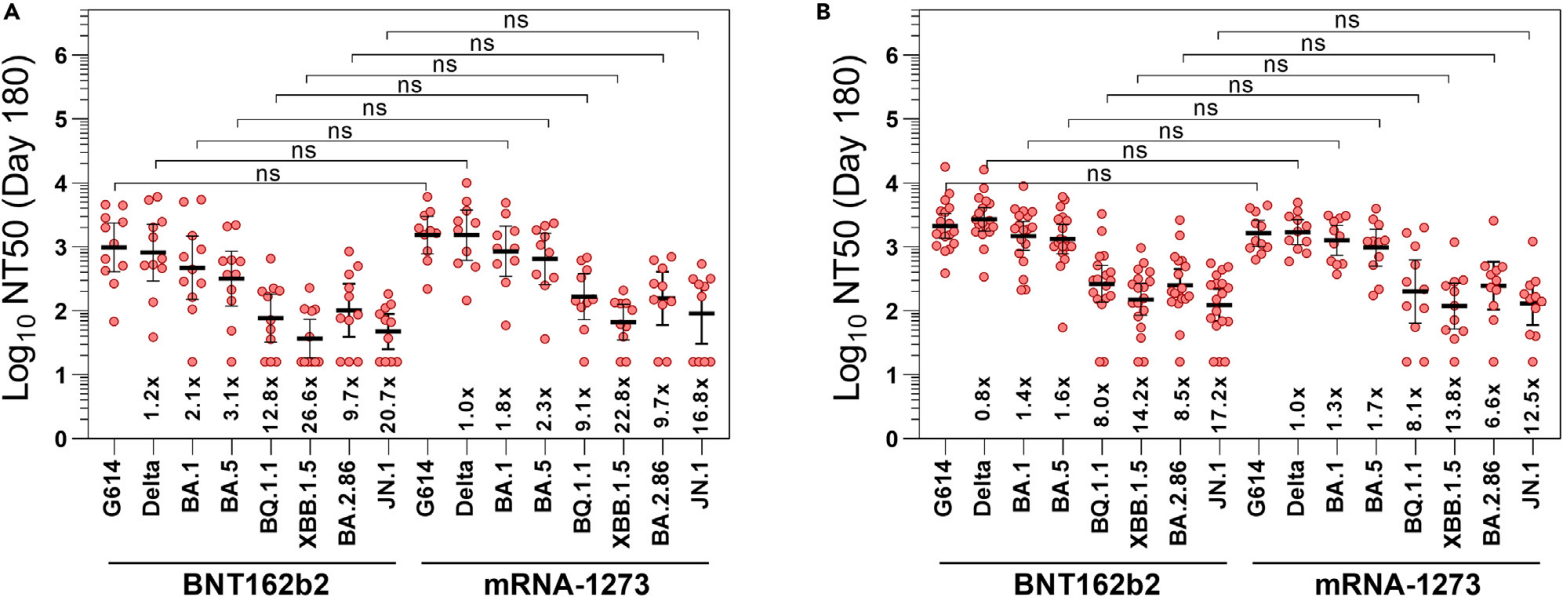
Neutralizing capacity to different SARS-CoV-2 variants at day 180 after the third dose (A and B) NT_50_ in pseudovirus neutralization assay against SARS-CoV-2 variants G614, Delta, and BA.1, BA.4/BA.5, BQ.1.1, XBB.1.5/XBB.1.9, BA.2.86, and JN.1 Omicron sublineages in N- (A) and N+ (B) participants. NT_50_ were summarized as geometric mean and 95% confidence interval. Ns: *p* > 0.05; ∗*p* ≤ 0.05; ∗∗*p* ≤ 0.01; ∗∗∗*p* ≤ 0.001; ∗∗∗∗*p* ≤ 0.0001. See also Tables S11–S16.

In order to assay the neutralization capacity of more evolved Omicron sublineages, NT_50_ titers against BA.4/BA.5, BQ.1.1, XBB.1.5/XBB.1.9, BA.2.86, and JN.1 were assayed for both arms at day 180 after the third vaccine dose. Lower neutralizing antibody titers compared with previous variants were observed at day 180 for BA.4/BA.5 sublineages with NT_50_ values of 318 (95% CI 118–856), and especially for BQ.1.1, XBB.1.5/XBB.1.9, and JN.1 with NT_50_ values of 77 (95% CI 33–181), 37 (95% CI 18–74), and 47 (95% CI 25–89), respectively, in those N- participants who received a third vaccine dose of BNT162b2 (Figure 4A). Similar titers were reached in N- participants receiving mRNA-1273 as the third vaccine dose, with NT_50_ values against BA.4/BA.5, BQ.1.1. XBB.1.5/XBB.1.9, and JN.1 of 648 (95% CI 257–1636), 167 (95% CI 72–383), 67 (95% CI 35–127), and 91 (95% CI 31–269), respectively (Figure 4A). These values represent an increase of 2.0-, 2.2-, 1.8-, and 1.9-fold in the neutralizing capacity of BA.4/BA.5, BQ.1.1, XBB.1.5/XBB.1.9, and JN.1, respectively, in mRNA-1273 arm compared with BNT162b2 arm, but differences were not statistically significant (Table S11).

Comparing N- and N+ participants vaccinated with BNT162b2 at day 180, a 4.2-, 3.4-, 4.1-, and 2.6-fold increases were observed in the NT_50_ titers against BA.4/BA.5, BQ.1.1, XBB.1.5/XBB.1.9, and JN.1, respectively in N+ individuals. On mRNA-1273 arm, the increases observed in asymptomatic participants with a recent SARS-CoV-2 infection in the neutralization capacity against BA.4/BA.5, BQ.1.1, XBB.1.5/XBB.1.9, and JN.1 were slightly lower, with rises of 1.6-, 1.3, 1.7-, and 1.4-fold compared with N- individuals (Figure 4; Table S12).

The proportion of N- participants exhibiting suboptimal neutralizing titers (NT_50_ < 100) at day 180 after the third dose with BNT162b2 was 9.1% for the ancestral G614, Delta and BA.1 Omicron variants, 18.2% for BA4/BA.5, 54.5% for BQ.1.1 and BA.2.86, and 72.7% for XBB.1.5/XBB.1.9 and JN.1 (Table S13). These proportions are lower for people who received mRNA-1273 vaccine as the third dose, with all the participants fully protected against G614, and Delta variants, and a suboptimal neutralizing response in 10% for BA.1 and BA.4/BA.5 Omicron sublineages, 20% for BQ.1.1 and BA.2.86, 40% for JN.1, and 50% for XBB.1.5/XBB.1.9 (Table S14). In the case of N+ participants with a recent history of SARS-CoV-2 infection, all the individuals reached NT_50_ values over 100 against G614, Delta, and BA.1 Omicron variants regardless of the mRNA vaccine received (Tables S15 and S16). A suboptimal neutralizing response was observed in BNT162b2 arm in a 5.5% for BA.4/BA.5, 11.1% for BQ.1.1 and BA.2.86, 27.8% for XBB.1.5/XBB.1.9, and 44.4% for JN.1 (Table S15) whereas these values in mRNA-1273 arm were 0% for BA.4/BA.5, 27.3% for BQ.1.1, BA.2.86, and JN.1, and 45.5% for XBB.1.5/XBB.1.9 (Table S16).

## Discussion

Although COVID-19 vaccine effectiveness in preventing SARS-CoV-2 infection and severe disease has been demonstrated for both adenoviral and mRNA-based vaccines,^17^^,^^18^ the capacity of mRNA vaccines to generate higher neutralizing responses is stronger compared with adenoviral-based vaccines.^19^ Interestingly, heterologous regimens based on the combination of both types of vaccines generates higher T cell responses,^19^ which are key for the clearance of the virus infection and control disease severity.^20^ A recent study associates heterologous schedules to a higher susceptibility to Omicron breakthrough infection, and also to stronger cellular responses than people with three doses of BNT162b2.^15^ This fact is especially relevant in the scenario observed in 2023 where several Omicron sublineages have become dominant and have replaced the previous ones by accumulating different mutations in different proteins of the virus. These mutations observed in emerging variants such as XBB sublineages and BA.2.86-derived variants (XBB.1.5, XBB.1.9, or XBB.1.16) could compromise the neutralizing response against the virus, but seem to have a limited effect on altering the T cell response.^21^ However, both humoral and cellular immune responses wane from natural SARS-CoV-2 infection and after vaccination, suggesting the need of studies about booster vaccinations following different schedules.^22^^,^^23^

In the present study, the third mRNA vaccine dose with either BNT162b2 or mRNA-1273 produces higher absolute values of SARS-CoV-2-specific anti-RBD total Igs at days 28, 90, and 180, compared with the immunogenicity reached any time after completing the ChAdOx1-S/BNT162b2 regimen, suggesting an optimal boost effect following the third dose. Other studies point that the immunogenicity of heterologous schedules based on two doses of ChAdOx1-S and one dose of BNT162b2 vaccines is slightly lower compared with three doses of BNT162b2, with drops of SARS-CoV-2 anti-spike IgGs of 8.3%–11.1%.^19^^,^^24^ Lower binding and neutralizing antibody titers were also observed in individuals vaccinated with ChAdOx1-S/BNT162b2/BNT162b2 regimen in comparison with three doses of BNT162b2.^15^ Heterologous schedules using one dose of BNT162b2 and one dose of NVXCoV2373 also provide diminished immunogenicity than the homologous BNT162b2 regimen in 50-to-70-year-olds participants of Com-COV2 trial, but these results were reverted in adolescents aged 12 to 16, since NVXCoV2373/BNT162b2 schedule elicited the highest humoral and peak cellular immune responses in Com-COV3 trial.^24^^,^^25^ N- individuals vaccinated with mRNA-1273 reached higher values of total Igs to SARS-CoV-2 RBD at day 28 compared with those receiving BNT162b2 and higher decreases of SARS-CoV-2 RBD total Igs were observed in N- individuals vaccinated with BNT162b2 compared with the mRNA-1273 arm, with drops of 45.9% and 15.8% at day 180, respectively. Similar results were observed for the titers of neutralizing antibodies, with higher levels in participants vaccinated with mRNA-1273 compared with BNT162b2 arm at day 28 and against VOCs Delta and BA.1 sublineage of Omicron. These results suggest that individuals following the triple-heterologous vaccine schedule (ChAdOx1-S/BNT162b2/mRNA-1273) reached an improved humoral response compared with double-heterologous regimens (ChAdOx1-S/BNT162b2/BNT162b2). Other studies have shown that heterologous schedules with three doses are less effective to reach a long-lasting neutralizing response against Omicron compared with three mRNA-1273 doses, evidencing the recommendation of a third dose, especially with mRNA-1273, for people vaccinated with any dose of ChAdOx1-S.^26^

XBB sublineages of Omicron have caused new waves of infections and severe cases and deaths in populations with high vaccination rates and high proportions of individuals with hybrid immunity after SARS-CoV-2 infections.^5^^,^^6^^,^^7^^,^^8^ One explanation to this situation described in countries such as South Africa, Denmark, or Singapore in late 2022 is the immune escape of XBB.1.5 along with faster waning of antibody titers. A recent study has observed low titers of neutralizing capacity of XBB.1.5 in individuals with three mRNA vaccine doses and BA.4/BA.5 breakthrough infections,^27^ so it was presumable stronger escape for individuals primed with ChAdOx1-S. In the present study, almost half of participants with a recent history of SARS-CoV-2 infection (pre-XBB sublineages) showed suboptimal titers of neutralizing antibodies against XBB.1.5/XBB.1.9 in mRNA-1273 arm or against JN.1 in BNT162b2 arm after three vaccine doses. As expected, these proportions were higher in participants without a recent history of SARS-CoV-2 infection, with values between 50.0% and 72.7% depending on the third vaccine dose administered. Although BA.5 bivalent mRNA vaccines could counteract this effect by boosting neutralizing antibodies against some Omicron sublineages,^28^ the response against JN.1, XBB.1.5, XBB.1.5-F456L variants such as EG.5.1 and other BA.2.75-derived variants carrying L452R mutation such as CH.1.1 or CA.3.1 is weak or absent for a significant proportion of individuals.^27^^,^^29^ This information is consistent with a recent report based on a deep learning model indicating that XBB.1.5 and JN.1 have the highest predicted immune escape potential according to their structural and biophysical properties.^30^ These results reinforce the past WHO recommendation of new booster doses ideally by monovalent vaccines targeting XBB-sublineages and the new EMA recommendation to use newer candidate vaccines in 2024 against variants belonging to the BA.2.86/JN.1 family, especially in susceptible populations such as the elderly or immunosuppressed individuals.

### Limitations of the study

The main limitation of this tertiary analysis of the CombiVacs study is inherited from the original study where the control group received a second dose one month later than the intervention group. In this third study, participants from those two original groups were combined prior to the second randomization and before the administration of the third dose. However, we believe that possible existing differences in immunogenicity generated by the second dose have little or no effect on the responses measured after the third dose. This study also fails to explore other components of the adaptive immune response against SARS-CoV-2, such as the virus-specific CD4^+^ and IFN-γ-producing CD8^+^ T cells or the SARS-CoV-2 spike-specific memory B cells. Another obvious limitation is the number of vaccinees for each arm of the clinical trial, especially after the inclusion of the anti-nucleocapsid IgG to identify asymptomatic infections. However, as a counterpart we think that this information is useful and allowed the study of the dynamics of humoral immune response in subjects asymptomatically infected.

## Resource availability

### Lead contact

Further information and request for resources and reagents should be directed to and will be fulfilled by the lead contact, Javier García-Pérez (eoaz@isciii.es).

### Materials availability

All reagents generated in this study are available from the lead contact with a completed materials transfer agreement. We may require a payment and/or a completed materials transfer agreement if there is potential for commercial application. We are glad to share these reagents with reasonable compensation by requestor for its processing and shipping.

### Data and code availability

#### Data

All data reported in this paper will be shared by the lead contact upon request.

#### Code

This paper does not report original code.

Any additional information required to reanalyze the data reported in this paper is available from the lead contact upon request.

## Acknowledgments

Funded by 10.13039/501100004587Instituto de Salud Carlos III (ISCIII). A.M.B., A.J.C., J.O., and J.F. are members of the VACCELERATE (European Corona Vaccine Trial Accelerator Platform) Network, which aims to facilitate and accelerate the design and implementation of COVID-19 phase 2 and 3 vaccine trials. J.O. is a member of the INsTRuCT (Innovative Training in Myeloid Regulatory Cell Therapy) Consortium, a network of European scientists from academia and industry focused on developing innovative immunotherapies. This work is funded by 10.13039/501100004587Instituto de Salud Carlos III, a Spanish public body assigned to the Ministry of Science and Innovation that manages and promotes public clinical research related to public health. The Spanish Clinical Trials Platform is a public network funded by the 10.13039/501100004587Instituto de Salud Carlos III (grant numbers PTC20/00018 and PT17/0017), the State Plan for Research, Development, and Innovation 2013–16, the State Plan for Scientific and Technical Research and Innovation 2017–20, and the Subdirectorate General for Evaluation and Promotion of Research, 10.13039/501100004587Instituto de Salud Carlos III, cofinanced with 10.13039/501100008530FEDER funds. CombiVacS was designed under the umbrella of the VACCELERATE project. VACCELERATE and INsTRuCT received funding from the EU's Horizon 2020 Research and Innovation Programme (grant agreement numbers 101037867 and 860003). The Instituto de Salud Carlos III is the Spanish partner in the VACCELERATE project. This work is funded by 10.13039/501100004587Instituto de Salud Carlos III through grants PI19CIII/00004 and PI21CIII/00025, the COVID-19 FUND (grants COV20/00679 and COV20/00072) and 10.13039/501100023961CIBERINFEC, co-financed with FEDER funds.

## Author contributions

Trial conceptualization was done by C.B.-I., J.A., M.P.-O., A.M.B., A.J.C., J.F., and J.R.A. Data curation was developed by J.G.-P. and M.P.-O. Formal analysis was done by J.G.-P., D.L., A.G.C., and F.D.-F. C.B.-I., J.A., M.P.-O., A.M.B., A.J.C., and J.F. were responsible for funding acquisition. J.G.-P., A.M.B., M.P.-O., M.B., A.L., A.C., M.C.-O., P.J.-S., E.T.-T., E.C., R.C., J.R.A, J.A., A.J.C., and F.D.-F. were study investigators. J.G.-P., A.M.B., M.P.-O., A.P., L.C., M.C.-A., M.J.B., M.B., A.L., A.A.-R., E.A.-A., I.F.C., A.V., A.C., M.T.G.-C., M.C.-O., C.P.I., D.L., P.J.-S., S.P.-R., A.G.-C., E.T.-T., E.C., R.C., J.A., A.J.C., and F.D.-F. developed the methodology of the study. The project administration was done by J.G.-P., A.M.B., M.P.-O., J.A., and A.J.C. J.G.-P., A.M.B., M.P.-O., A.P., L.C., M.C.-A., M.J.B., M.B., J.R.A., A.A.-R., E.A.-A., I.F.C., A.V., M.T.G.-C., C.P.I., D.L., A.G.-C., J.A., A.J.C., and F.D.-F. provided the trial resources. J.G.-P., D.L., and F.D.-F. participated in the implementation of the software and in data visualization. The trial supervision was conducted by J.G.-P., A.M.B., M.P.-O., J.A., and A.J.C. J.G.-P., D.L., M.P.-O., and F.D.-F. ensured data validation. J.G.-P., M.P.-O., J.A., A.J.-C., and F.D.-F. wrote the original draft of this article. All authors reviewed and edited the manuscript, and approved the manuscript for submission. All authors had full access to the full data in the study and accept responsibility to submit for publication.

J.G.-P., D.L., and F.D.-F. performed statistical analyses. J.G.-P., M.P.-O., J.A., A.J.-C., and F.D.-F. had unrestricted access to all data. J.G.-P. and F.D.-F. prepared the original draft of the manuscript and all the authors reviewed it and edited it. All authors agreed to submit the manuscript, read, and approved the final draft and take full responsibility of its content, including the accuracy of the data and the fidelity of the trial to the registered protocol and its statistical analysis.

## Declaration of interests

J.A. has received fees for educational programs from Gilead, MSD, GSK and Janssen outside of the submitted work. M.C.-A. has participated in advisory boards and has received research funding from GSK, Sanofi Pasteur, Pfizer, Novavax, and Janssen. C.B.-I. is the deputy general manager of the Instituto de Salud Carlos III. J.R.A. has received fees from Janssen, outside of the submitted work. A.M.B. is principal investigator of clinical trials sponsored by GlaxoSmithKline, Daiichi-Sankyo, Janssen, and Farmalider, outside of the submitted work.

## STAR★Methods

### Key resources table

**Table:** 

REAGENT or RESOURCE	SOURCE	IDENTIFIER
**Biological samples**		

Blood from the 79 subjects included in the study from four time points (Days 0, 28, 90 and 180 post-vaccination)	La Paz University Hospital (Madrid), San Carlos Hospital (Madrid), Cruces University Hospital (Vizcaya), Vall d’Hebron University Hospital (Barcelona) and Clinic Hospital (Barcelona)	N/A

**Chemicals, peptides, and recombinant proteins**		

Codon-optimized spike DNA sequences for D614G, B.1.617.2, BA.1, BA.4/BA.5, BQ.1.1, XBB.1.5/XBB.1.9, BA.2.86, and JN.1 SARS-CoV-2 variants	GeneArt Gene Synthesis (ThermoFisher)	N/A

**Critical commercial assays**		

Elecsys® Anti-SARS-CoV-2 S	Roche Diagnostics	Cat#09203079190
BioPlex 2200 SARS-CoV-2 IgG	Bio-Rad Laboratories	Cat# 12014192
Renilla Luciferase Assay	Promega	Cat#E2820

**Experimental models: Cell lines**		

Vero E6	Laboratory of Dr. Antonio Alcamí	N/A
HEK-293T	NIBSC	N/A

**Recombinant DNA**		

pcDNA3.1D/V5-His-TOPO	Invitrogen™	Cat#K490001
pcDNA-VSV-G	Laboratory of Dr. Arenzana Seisdedos	N/A
pNL4-3ΔenvRen	García-Pérez et al., 2007^31^	N/A
pcDNA3.1-S-CoV2Δ19-G614	This paper	N/A
pcDNA3.1-S-CoV2Δ19-Delta	This paper	N/A
pcDNA3.1-S-CoV2Δ19-BA.1	This paper	N/A
pcDNA3.1-S-CoV2Δ19-BA.4-BA.5	This paper	N/A
pcDNA3.1-S-CoV2Δ19-BQ.1.1	This paper	N/A
pcDNA3.1-S-CoV2Δ19-XBB.1.5	This paper	N/A
pcDNA3.1-S-CoV2Δ19-BA.2.86	This paper	N/A
pcDNA3.1-S-CoV2Δ19-JN.1	This paper	N/A

**Software and algorithms**		

GraphPad Prism version 9.0.1	GraphPad Software, Inc.	N/A
R 4.3.2 and ggplot2 package	Free software	N/A

### Experimental model and study participant details

Data from the 24-month, phase 2, open-label, randomized, controlled CombiVacs study are included in this tertiary analysis. Full descriptions of the methods, safety and initial immunogenicity analyses as well as the durability of the immune response have been previously published in detail.^3^^,^^4^

All participants provided written informed consent before enrolment. The trial complies with the principles of the Declaration of Helsinki and Good Clinical Practice. This study was approved by the Ethics Committee at University Hospital La Paz and by the Spanish Agency of Medicines and Healthcare Products (AEMPS) on April 19, 2021 (protocol version 1.1 15Apr2021). The first substantial amendment for the second part of third vaccine dose was approved by Ethics Committee and AEMPS on December 30, 2021 (protocol version 2.0 12Dec2021). A total of 13 (44.8%) participants were woman in BNT162b2 arm and 12 (57.1%) in mRNA-1273 arm. In BNT162b2 arm, 14 participants (48.3%) were ages 18-49 years old and 15 participants (51.7%) were ages 50-59 years old. In mRNA-1273 arm, 11 participants (52.4%) were ages 18-49 years old and 10 (47.6%) were ages 50-59 (Table 1).

The Vero E6 (African green monkey kidney) cell line was kindly provided by Dr. A. Alcami (CBM Severo Ochoa, Madrid). Vero E6 cells were cultured in DMEM supplemented with 10% FCS, 2 mM L-glutamine and 100 units/ml penicillin and streptomycin (Lonza).

HEK-293T were obtained from the National Institute for Biological Standards and Control (NIBSC). HEK-293T cells were cultured in DMEM supplemented with 10% FCS, 2 mM L-glutamine and 100 units/ml penicillin and streptomycin (Lonza).

### Method details

#### Study design and population

A total of 676 participants primed with ChAdOx1-S were enrolled and randomized in the CombiVacS clinical trial. Among them, 450 participants were assigned to receive a BNT162b2 boost and 418 completed the visits at days 14, 28, 90, and 180 post-vaccination. Subjects that have completed all the previous visits (6 months after the first prime-boost vaccination with ChAdOx1-S/BNT162b2) enrolled for the second part of third vaccine dose.

#### Randomization and masking

Subjects that have completed visit 6 (six months after randomization in Part 1), and that have completed a prime-boost vaccination with ChAdOx1-S/BNT162b2 were randomized to receive a third dose of a mRNA vaccine (BNT162b2 or mRNA-1273). Subjects were randomized in a proportion of 1:1 and stratified by study site and age. Subjects that have received a third dose before randomization, will be able to make follow-up visits within the study. No blinding was planned in this open-label trial.

All subjects included in the first randomization will be included in the second part, therefore no sample size calculation is needed for the present study.

#### Procedures

All participants included in this tertiary analysis received a third vaccine dose with a mRNA-based vaccine, either a BNT162b2 dose administered at the approved dose of 30 μgor a half dose (50 μg) of mRNA-1273, both as a single intramuscular injection at day 0 of the present study (which coincide with 12 months after the second vaccine dose). Follow-up visits were scheduled on days 28, 90, and 180 after the third dose to record medical and medication history and to collect blood samples for immune response evaluation.

#### SARS-CoV-2 specific humoral immune response

IgG to SARS-CoV-2 nucleocapsid was quantified using the BioPlex 2200 SARS-CoV-2 IgG assay (Bio-Rad Laboratories) according to the manufacturer’s instructions coupled with BioPlex 2200 SARS-CoV-2 IgG Calibrators diluted in BioPlex 2200 Wash Buffer (Bio-Rad) using a 1:8 ratio. A result of < 23 BAU/mL and ≥ 24 BAU/mL was interpreted as negative and positive, respectively.

SARS-CoV-2-specific humoral immune response was measured with the commercial Elecsys Anti-SARS-CoV-2 electrochemiluminiscent immunoassay (Roche Diagnostic) to test for total immunoglobulins to SARS-CoV-2 RBD spike on the Cobas e411 module (Roche Diagnostic). Measuring ranged from 0.4 U/mL to 250.0 U/mL (up to 2500 U/mL with onboard 1:10 dilution, and up to 12 500 U/mL with onboard 1:50 dilution). Values higher than 0.8 BAU/mL were considered positive (the correlation between U/mL and BAU/mL was 1 U/ml = 0.972 BAU/ml).

#### SARS-CoV-2 pseudovirus neutralization assay

SARS-CoV-2 pseudovirus neutralization assay was used to quantify neutralizing antibody titers specific for Delta and different Omicron lineages (BA.1, BA.4/BA.5, BQ.1.1, XBB.1.5/XBB.1.9, BA.2.86, and JN.1). The codon-optimized sequence was modified by synthesis (GeneArt Gene Synthesis, ThermoFisher Scientific) to generate the different spikes of SARS-CoV-2 virus: D614G (as the reference variant), Delta (lineage B.1.617.2), and the Omicron lineages BA.1, BA.4/BA.5, BQ.1.1, XBB.1.5/XBB.1.9, BA.2.86, and JN.1 by including the mutations showed in Figure S3.^4^ All these sequences were synthesized removing the last 19 amino acids and were inserted in pcDNA3.1D/V5-His-TOPO between BamHI and XbaI sites. The pcDNA-VSV-G plasmid contains the cDNA encoding the vesicular stomatitis virus G protein and was obtained from Dr. Arenzana-Seisdedos (Institute Pasteur, Paris, France). VSV-G pseudoviruses were used as a control of specificity in neutralization testing.

NL4.3 pseudotypes were generated with the plasmid pNL4-3ΔenvRen. Briefly, Renilla luciferase reporter pseudovirus were prepared by co-transfecting HEK-293T cells with pNL4-3ΔenvRen backbone and viral envelope protein expression plasmid pcDNA3.1-S-CoV2Δ19 or pcDNA-VSV-G using the calcium phosphate method. The medium was changed 18 hours after transfection, and 48 hours post-transfection cell culture supernatants were harvested, clarified by centrifugation at 500 x g for 5 min, and frozen at -80°C. The amount of HIV p24 antigen in the supernatants was quantified by electrochemiluminescence Immunoassay (Roche Diagnostic) on the Cobas e411 module (Roche Diagnostic).

To measure neutralizing activity of plasma from donors, four-fold serial dilutions of heat-inactivated sera (1:32-1:131072) were preincubated with titrated pseudoviruses (10 ng of p24 Gag/well) for 1 hour at 37°C. Thereafter, 100 μl of the mixture was added to Vero E6 cells plated the previous day at 5 x 10^3^ cells/well in 100 μl medium in 96-well plates. The culture medium was refreshed after 16 hours. At 48 h post-infection, cells were lysed, and viral infectivity was assessed by measuring luciferase activity (Renilla Luciferase Assay, Promega, Madison, WI, USA) using a 96-well plate luminometer LB 960 Centro XS³ (Berthold Technologies, Oak Ridge,TN, USA). The detection threshold for the assay is 1:32 serum dilution. Samples below this threshold received a value of 1:16. The titers of neutralizing antibodies were calculated as 50% inhibitory dose (neutralizing titer 50, NT_50_), expressed as the highest dilution of plasma which resulted in a 50% reduction of luciferase activity compared to control without plasma. Sigmoid curves were generated and NT_50_s were calculated by non-linear regression using GraphPad Prism version 9.3.1 (GraphPad Software, Inc.).

### Quantification and statistical analysis

All analyses will be carried out using the statistical software SAS, version 9.4 of the SAS system for Windows.

All continuous variables were summarized using the following descriptive statistics: n (non-missing sample size), mean, standard deviation (SD), 1st quartile, median, 3rd quartile, maximum and minimum. Absolute and relative frequencies (based on the non-missing sample size) of observed levels were reported for all categorical variables.

All continuous variables were tested for normality hypothesis assumption with the Kolmogorov-Smirnov test. The antibodies binding to the SARS-CoV-2 S protein were measured as lognormal distribution to obtain more nearly symmetric distribution.

Baseline data were collected just before intervention at day 0. Participants with missing baseline value were excluded from the analysis.

The immune response measured by immunoassays at baseline and days 28, 90, and 180 after randomization between the two arms was tested by two-tailed t-test assuming symmetric distribution of lognormal transformation. Geometric means were calculated as the mean of the assay results after making the logarithm transformation and then exponentiating the mean to express results on the original scale. Two-sided 95% CIs were obtained by taking natural log transforms of concentrations/titers, calculating the 95% CI with reference to the t-distribution, and then exponentiating the confidence limits. In other case, Mann-Whitney-Wilcoxon test was used.

### Additional resources

The clinical trial registry numbers of the CombiVacs study are 2021-001978-37 in EudraCT (https://www.clinicaltrialsregister.eu/ctr-search/trial/2021-001978-37/ES) and NCT04860739 in ClinicalTrials.gov (https://clinicaltrials.gov/).
