# Supplementary material for: Immunogenicity of a third dose with mRNA-vaccines in the ChAdOx1-S/BNT162b2 vaccination regimen against SARS-CoV-2 variants
Source: iScience. 2024 Aug 14;27(9):110728. doi: 10.1016/j.isci.2024.110728 (PMC11404211; doi:10.1016/j.isci.2024.110728)
Supplement: Document S1. Figures S1–S3 and Tables S1–S16 [file mmc1.pdf]

iScience, Volume *27*

Supplemental information

Immunogenicity of a third dose with mRNA-vaccines in the ChAdOx1-S/BNT162b2 vaccination regimen against SARS-CoV-2 variants

Javier García-Pérez, Alberto M. Borobia, Mayte Pérez-Olmeda, Antonio Portolés, Luis Castaño, Magdalena Campins-Artí, María Jesús Bertrán, Mercedes Bermejo, José Ramón Arribas, Andrea López, Ana Ascaso-del-Rio, Eunate Arana-Arri, Inmaculada Fuentes Camps, Anna Vilella, Almudena Cascajero, María Teresa García-Morales, María Castillo de la Osa, Carla Pérez Ingidua, David Lora, Paloma Jiménez-Santana, Silvia Pino-Rosa, Agustín Gómez de la Cámara, Erick De La Torre-Tarazona, Esther Calonge, Raquel Cruces, Cristóbal Belda-Iniesta, José Alcamí, Jesús Frías, Antonio J. Carcas, Francisco Díez-Fuertes, and CombiVacS Study Group

Data S1. Figures S1-S3 and Tables S1-S16.

Figure S1. Humoral immune response by age, related to figures 2 and 4. Group A represents participants aged 18-49 years and group B those aged 50-59 years. Total anti-RBD Igs were expressed as BAU/ml and variant-specific neutralization capacity as NT50. Data are represented as median and interquartile range.

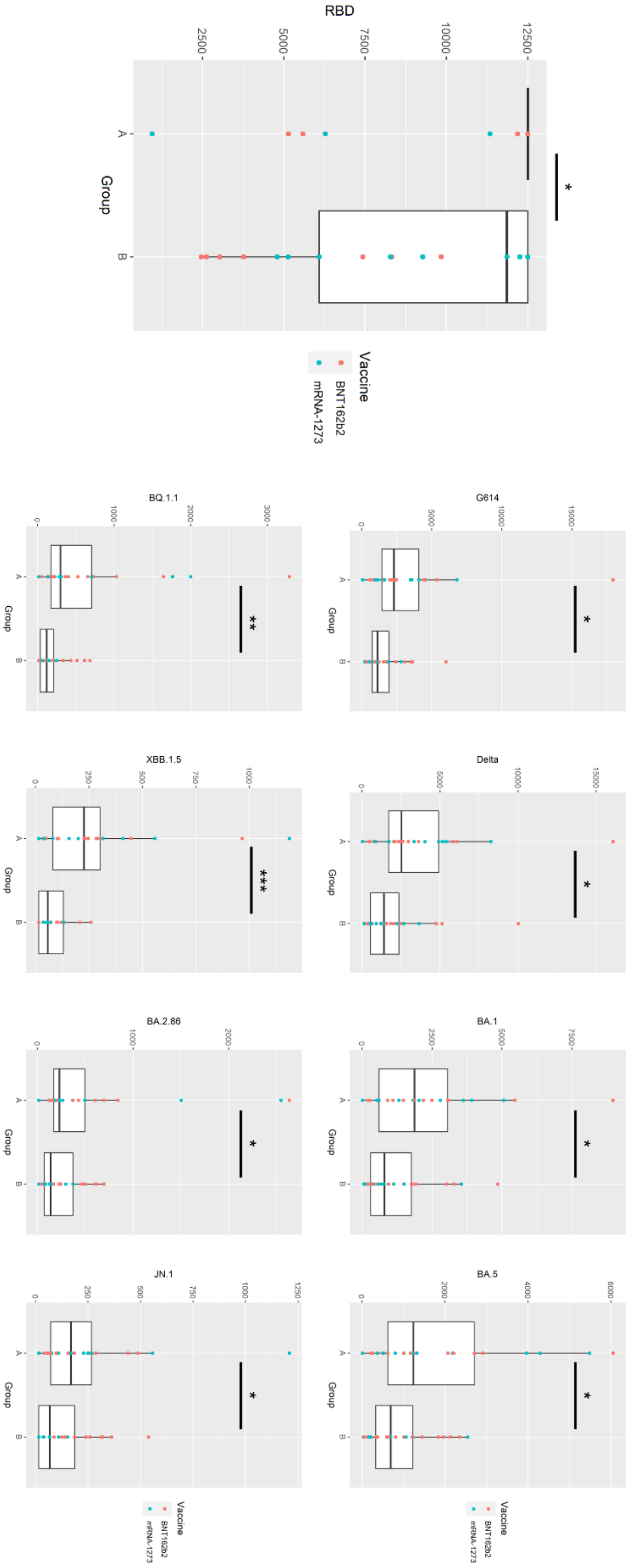

Figure S2. Humoral immune response by sex, related to figures 2 and 4. Total anti-RBD Igs were expressed as BAU/ml and variant-specific neutralization capacity as NT50. Data are represented as median and interquartile range.

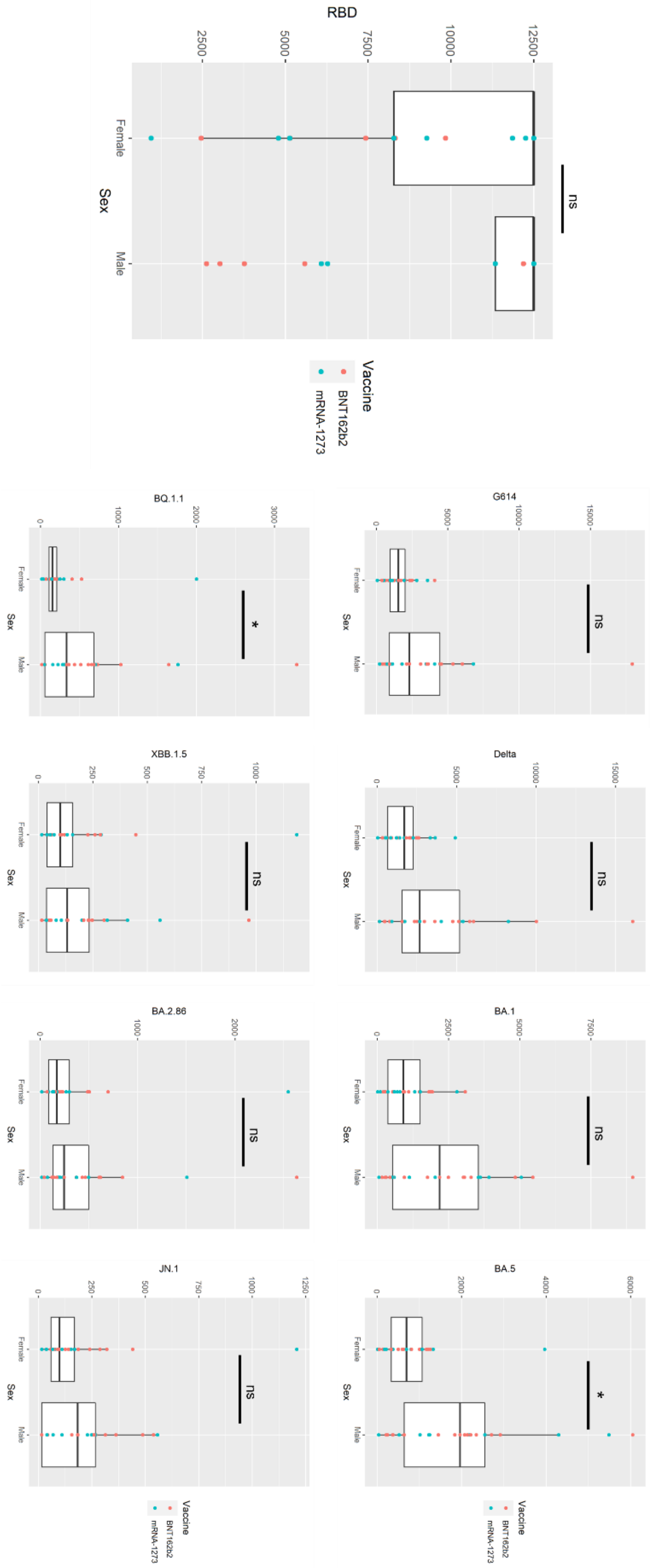

Figure S3. Heatmap showing the prevalence of the mutations of the lineages used in this study according to GISAID database, related to STAR Methods.

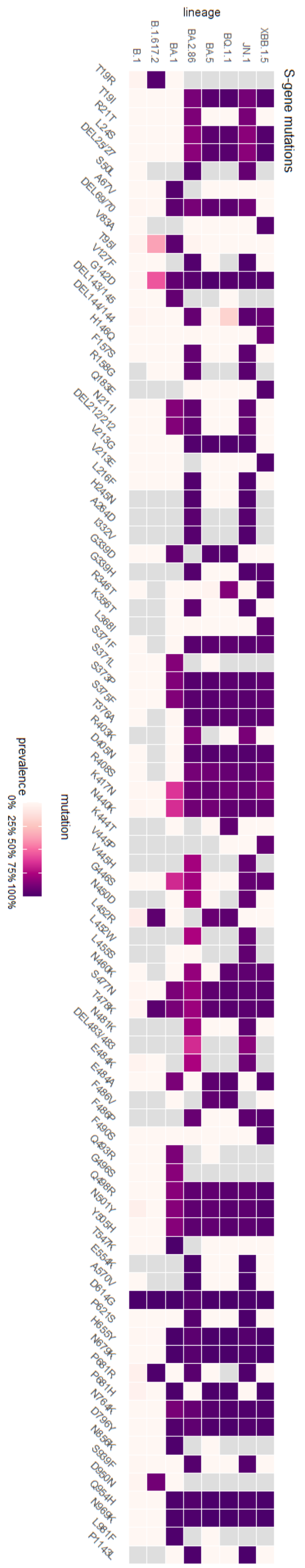

Table S1. Summary statistics for SARS-CoV-2 anti-spike Ig response comparing BNT162b2 and mRNA-1273 arms in participants with a negative result of IgG to SARS-CoV-2 nucleocapsid, related to Figure 2.

	BNT162b2		mRNA-1273		p-value
	n	Geometric mean [95% CI]	n	Geometric mean [95% CI]	
**day 0**	33	1381.9[990.0;1929.0]	28	1154.7[754.3;1767.6]	0.307
**day 28**	30	12220.6[11896.8;12553.1]	26	12243.3[11861.7;12637.2]	0.903
**day 90**	24	9157.7[7813.6;10733.1]	22	10431.8[9158.3;11882.3]	0.184
**day 180**	11	6613.1[3687.5;11859.9]	10	10302.9[8156.7;13013.8]	0.138

Units: BAU/mL

Table S2. Comparative neutralization of SARS-CoV-2 ancentral G614 variant in BNT162b2 and mRNA-1273 arms in participants with a negative result of IgG to SARS-CoV-2 nucleocapsid, related to Figure 2.

	BNT162b2		mRNA-1273		p-value
	n	Geometric mean [95% CI]	n	Geometric mean [95% CI]	
**day 0**	33	113.7[75.0;172.4]	28	129.4[73.0;229.5]	0.728
**day 28**	30	1929.0[1499.7;2481.3]	26	2693.3[2025.8;3580.7]	0.080
**day 90**	24	1010.7[707.3;1444.3]	22	1794.9[1223.9;2632.4]	0.038
**day 180**	11	980.2[405.9;2367.0]	10	1522.0[771.7;3002.0]	0.597

Units: neutralising titer 50 (NT_{50})

Table S3. Comparative neutralization of SARS-CoV-2 Delta variant (B.1.617.2 lineage) in BNT162b2 and mRNA-1273 arms in participants with a negative result of IgG to SARS-CoV-2 nucleocapsid, related to Figure 2.

	BNT162b2		mRNA-1273		p-value
	n	Geometric mean [95% CI]	n	Geometric mean [95% CI]	
**day 0**	33	74.0[45.5;120.4]	28	116.1[63.8;211.3]	0.330
**day 28**	30	2027.1[1539.5;2669.1]	26	2926.7[2196.2;3900.0]	0.022
**day 90**	24	852.3[538.6;1348.7]	22	1500.2[934.2;2409.3]	0.073
**day 180**	11	811.8[291.7;2259.1]	10	1525.1[615.4;3779.6]	0.342

Units: neutralising titer 50 (NT_{50})

Table S4. Comparative neutralization of SARS-CoV-2 Omicron variant (BA.1 lineage) in BNT162b2 and mRNA-1273 arms in participants with a negative result of IgG to SARS-CoV-2 nucleocapsid, related to Figure 2.

	BNT162b2		mRNA-1273		p-value
	n	Geometric mean [95% CI]	n	Geometric mean [95% CI]	
**day 0**	33	36.1[22.4;58.3]	28	42.2[22.2;80.1]	0.839
**day 28**	30	725.0[493.4;1065.2]	26	1368.3[934.9;2002.6]	0.009
**day 90**	24	508.4[327.0;790.5]	22	904.9[534.4;1532.4]	0.106
**day 180**	11	471.1[150.4;1475.6]	10	852.4[344.3;2110.2]	0.342

Units: neutralising titer 50 (NT_{50})

Table S5. Summary statistics for SARS-CoV-2 anti-spike Ig response comparing BNT162b2 and mRNA-1273 arms in participants with a positive result of IgG to SARS-CoV-2 nucleocapsid, related to Figure 2.

	BNT162b2		mRNA-1273		p-value
	n	Geometric mean [95% CI]	n	Geometric mean [95% CI]	
**day 0**	8	9764.0[6851.6;13914.5]	6	9630.4[6148.9;15083.2]	0.830
**day 28**	10	12326.2[11942.0;12722.8]	7	12500[12500;12500]	0.473
**day 90**	13	10685.5[8788.5;12992.0]	9	11631.8[10324.9;13104.1]	0.640
**day 180**	18	9567.8[7467.6;12258.8]	11	10755.2[8739.9;13235.2]	0.652

Units: BAU/mL

Table S6. Comparative neutralization of SARS-CoV-2 ancestral G614 variant in BNT162b2 and mRNA-1273 arms in participants with a positive result of IgG to SARS-CoV-2 nucleocapsid, related to Figure 2.

	BNT162b2		mRNA-1273		p-value
	n	Geometric mean [95% CI]	n	Geometric mean [95% CI]	
**day 0**	8	1530.9[709.0;3305.3]	6	1054.1[436.4;2546.4]	0.561
**day 28**	10	4458.6[3199.8;6212.5]	7	5192.5[2981.1;9044.2]	0.526
**day 90**	13	2435.5[1494.1;3970.0]	9	3422.3[1558.0;7517.1]	0.841
**day 180**	18	2123.9[1363.7;3307.6]	11	1624.1[1015.8;2596.7]	0.432

Units: neutralising titer 50 (NT_{50})

Table S7. Comparative neutralization of SARS-CoV-2 Delta variant (B.1.617.2 lineage) in BNT162b2 and mRNA-1273 arms in participants with a positive result of IgG to SARS-CoV-2 nucleocapsid, related to Figure 2.

	**BNT162b2**		**mRNA-1273**		**p-value**
	n	Geometric mean [95% CI]	n	Geometric mean [95% CI]	
**day 0**	8	2203.0[1227.3;3954.4]	6	1239.9[492.0;3124.9]	0.175
**day 28**	10	5512.8[4261.2;7132.1]	7	5347.3[2973.3;9616.7]	0.733
**day 90**	13	2633.7[1818.2;3815.2]	9	2897.0[1922.1;4366.2]	0.841
**day 180**	18	2694.9[1762.9;4119.7]	11	1699.5[1082.1;2669.0]	0.157

Units: neutralising titer 50 (NT_{50})

Table S8. Comparative neutralization of SARS-CoV-2 Omicron variant (BA.1 lineage) in BNT162b2 and mRNA-1273 arms in participants with a positive result of IgG to SARS-CoV-2 nucleocapsid, related to Figure 2.

	BNT162b2		mRNA-1273		p-value
	n	Geometric mean [95% CI]	n	Geometric mean [95% CI]	
**day 0**	8	1390.8[738.8;2618.1]	6	1123.9[395.7;3192.2]	0.948
**day 28**	10	3019.9[1963.0;4645.9]	7	3338.2[1807.1;6166.9]	0.733
**day 90**	13	1825.2[1150.7;2895.0]	9	1634.4[1052.2;2538.6]	0.504
**day 180**	18	1480.3[880.8;2487.9]	11	1258.3[735.5;2152.8]	0.544

Units: neutralising titer 50 (NT_{50})

Table S9. Comparative humoral immune response against SARS-CoV-2 variants G614, Delta, and BA.1 between participants with a negative and positive result of IgG to SARS-CoV-2 nucleocapsid in BNT162b2 arm, related to Figure 2.

		N-*		N+**		p-value
		n	Geometric mean [95% CI]	n	Geometric mean [95% CI]	
**Day 0**	**RBD-Igs**	33	1381.9 [990.0; 1929.0]	8	9764.0 [6851.6- 13914.5]	6.3×10^{-5}
	**G614**	33	113.7 [75.0; 172.4]	8	1530.9 [709.0; 3305.3]	5.6×10^{-5}
	**Delta**	33	74.0 [45.5; 120.4]	8	2203.0 [1227.3; 3954.4]	3.1×10^{-5}
	**BA.1**	33	36.1 [22.4; 58.3]	8	1390.8 [738.8; 2618.1]	1.6×10^{-5}
**Day 28**	**RBD-Igs**	30	12220.6 [11896.8; 12553.2]	10	12326.2 [11942.0; 12722.8]	0.952
	**G614**	30	1929.0 [1499.7; 2481.3]	10	4458.6 [3199.8; 6212.5]	4.3×10^{-4}
	**Delta**	30	2027.1 [1539.5; 2669.1]	10	5512.8 [4261.2; 7132.1]	4.0×10^{-5}
	**BA.1**	30	725.0 [493.4; 1065.2]	10	3019.9 [1963.0; 4645.9]	4.0×10^{-5}
**Day 90**	**RBD-Igs**	24	9157.7 [7813.6; 10733.1]	13	10685.5 8788.5; 12992.0]	0.1046
	**G614**	24	1010.7 [707.3; 1444.3]	13	2435.5 [1494.1; 3970.0]	0.004
	**Delta**	24	852.3 [538.6; 1348.7]	13	2633.8 [1818.2; 3815.2]	3.2×10^{-4}
	**BA.1**	24	508.4 [327.0; 790.5]	13	1825.2 [1150.7; 2895.0]	5.0×10^{-4}
**Day 180**	**RBD-Igs**	11	6613.1 [3687.5; 11859.9]	18	9567.8 [7467.6; 12258.8]	0.092
	**G614**	11	980.2 [405.9; 2367.0]	18	2123.9 [1363.8; 3307.7]	0.204
	**Delta**	11	811.8 [291.7; 2259.1]	18	2694.9 [1762.9; 4119.7]	0.032
	**BA.1**	11	471.1 [150.4; 1475.6]	18	1480.4 [880.8; 2487.9]	0.061

Units: neutralising titer 50 (NT_{50})
*N-: negative result of IgG to SARS-CoV-2 nucleocapsid
**N+: positive result of IgG to SARS-CoV-2 nucleocapsid

Table S10. Comparative humoral immune response against SARS-CoV-2 variants G614, Delta, and BA.1 between participants with a negative and positive result of IgG to SARS-CoV-2 nucleocapsid in mRNA-1273 arm, related to Figure 2.

		N-*		N+**		p-value
		n	Geometric mean [95% CI]	n	Geometric mean [95% CI]	
**Day 0**	**RBD-Igs**	28	1154.7 [754.3; 1767.6]	6	9630.5 [6148.9; 15083.2]	7.4 x 10-4
	**G614**	28	129.4 [73.0; 229.5]	6	1054.1 [436.4; 2546.4]	0.003
	**Delta**	28	116.1 [63.8; 211.3]	6	1239.9 [492.0; 3124.9]	0.002
	**BA.1**	28	42.2 [22.2; 80.1]	6	1123.9 [395.7; 3192.2]	9.1 x 10-4
**Day 28**	**RBD-Igs**	26	12243.3 [11861.7; 12637.2]	7	12500.0 [12500.0; 12500.0]	0.377
	**G614**	26	2693.3 [2025.8; 3580.7]	7	5192.5 [2981.2; 9044.2]	0.043
	**Delta**	26	2926.7 [2196.2; 3900.0]	7	5347.3 [2973.3; 9616.7]	0.099
	**BA.1**	26	1368.3 [934.9; 2002.6]	7	3338.3 [1807.1; 6166.9]	0.043
**Day 90**	**RBD-Igs**	22	10431.8 [9158.3; 11882.3]	9	11631.8 [10324.9; 13104.1]	0.244
	**G614**	22	1795.0 [1223.9; 2632.4]	9	3422.3 [1558.0; 7517.1]	0.094
	**Delta**	22	1500.2 [934.2; 2409.3]	9	2897.0 [1922.1; 4366.2]	0.023
	**BA.1**	22	904.9 [534.4; 1532.4]	9	1634.4 [1052.2; 2538.6]	0.078
**Day 180**	**RBD-Igs**	10	10302.9 [8156.7; 13013.8]	11	10755.2 [8740.0; 13235.2]	0.717
	**G614**	10	1522.0 [771.7; 3002.0]	11	1624.1 [1015.8; 2596.7]	0.918
	**Delta**	10	1525.1 [615.4; 3779.6]	11	1699.5 [1082.1; 2669.0]	0.972
	**BA.1**	10	852.4 [344.3; 2110.2]	11	1258.3 [735.5; 2152.8]	0.704

Units: neutralising titer 50 (NT_{50})
*N-: negative result of IgG to SARS-CoV-2 nucleocapsid
**N+: positive result of IgG to SARS-CoV-2 nucleocapsid

Table S11. Comparative neutralization of SARS-CoV-2 Omicron sublineages (BA4/BA.5, BQ.1.1, and XBB.1.5/XBB.1.9) in BNT162b2 and mRNA-1273 arms at day 180, related to Figure 4.

		BNT162b2		mRNA-1273		p-value
		n	Geometric mean [95% CI]	n	Geometric mean [95% CI]	
N-*	**BA.4/BA.5**	11	317.7 [117.9; 855.8]	10	648.4 [257.0; 1636.1]	0.245
	**BQ.1.1**	11	76.8 [32.5; 181.5]	10	166.6 [72.4; 383.1]	0.289
	**XBB.1.5/XBB.1.9**	11	36.9 [18.4; 74.0]	10	66.8 [35.1; 127.1]	0.180
	**BA.2.86**	11	101.5 [39.1; 263.2]	10	156.4 [59.8; 408.9]	0.457
	**JN.1**	11	47.3 [25.0; 89.4]	10	90.6 [30.6; 268.6]	0.205
N+**	**BA.4/BA.5**	18	1327.8 [778.8; 2263.7]	11	970.1 [502.3; 1873.6]	0.514
	**BQ.1.1**	18	263.9 [136.6; 510.0]	11	199.6 [64.2; 620.7]	0.574
	**XBB.1.5/XBB.1.9**	18	150.1 [84.6; 266.1]	11	117.9 [51.8; 268.3]	0.458
	**BA.2.86**	18	249.9 [137.7; 453.4]	11	245.7 [103.8; 581.7]	0.928
	**JN.1**	18	123.3 [68.9; 220.7]	11	129.6 [59.8; 281.2]	0.822

Units: neutralising titer 50 (NT_{50})
*N-: negative result of IgG to SARS-CoV-2 nucleocapsid
**N+: positive result of IgG to SARS-CoV-2 nucleocapsid

Table S12. Comparative neutralization of SARS-CoV-2 Omicron sublineages (BA4/BA.5, BQ.1.1, and XBB.1.5/XBB.1.9) between N- and N+ subjects in BNT162b2 and mRNA-1273 arms at day 180, related to Figure 4.

		N-*		N+**		p-value
		n	Geometric mean [95% CI]	n	Geometric mean [95% CI]	
**BNT162b2**	**BA.4/BA.5**	11	317.7 [117.9; 855.8]	10	1327.8 [778.8; 2263.7]	0.004
	**BQ.1.1**	11	76.8 [32.5; 181.5]	10	263.9 [136.6; 510.0]	0.026
	**XBB.1.5/XBB.1.9**	11	36.9 [18.4; 74.0]	10	150.1 [84.6; 266.1]	0.005
	**BA.2.86**	11	101.5 [39.1; 263.2]	10	249.9 [137.7; 453.4]	0.137
	**JN.1**	11	47.3 [25.0; 89.4]	10	123.3 [68.9; 220.7]	0.032
**mRNA-1273**	**BA.4/BA.5**	18	648.4 [257.0; 1636.1]	11	970.1 [502.3; 1873.6]	0.605
	**BQ.1.1**	18	166.6 [72.4; 383.1]	11	199.6 [64.2; 620.7]	0.860
	**XBB.1.5/XBB.1.9**	18	66.8 [35.1; 127.1]	11	117.9 [51.8; 268.3]	0.341
	**BA.2.86**	18	156.4 [59.8; 408.9]	11	245.7 [103.8; 581.7]	0.647
	**JN.1**	18	90.6 [30.6; 268.6]	11	129.6 [59.8; 281.2]	0.972

Units: neutralising titer 50 (NT_{50})
*N-: negative result of IgG to SARS-CoV-2 nucleocapsid
**N+: positive result of IgG to SARS-CoV-2 nucleocapsid

Table S13. Neutralizing response by NT_{50} categories against different SARS-CoV-2 variants in N- subjects of BNT162b2 arm at day 180, related to Figure 4.

	BNT162b2 (n=11)															
	G614		Delta		BA.1		BA.5		BQ.1.1		XBB.1.5		BA.2.86		JN.1	
	n	%	n	%	n	%	n	%	n	%	n	%	n	%	n	%
**<100, n (%)**	1	9.1	1	9.1	1	9.1	2	18.2	6	54.5	8	72.7	6	54.5	8	72.7
**>100 to <300, n (%)**	1	9.1	1	9.1	4	36.4	2	18.2	4	36.4	3	27.3	2	18.2	3	27.3
**>300 to <1000, n (%)**	3	27.3	4	36.4	3	27.3	5	45.5	1	9.1	0	0.0	3	27.3	0	0.0
**≥1000, n (%)**	6	54.5	5	45.5	3	27.3	2	18.2	0	0.0	0	0.0	0	0.0	0	0.0

Table S14. Neutralizing response by NT_{50} categories against different SARS-CoV-2 variants in N- subjects of mRNA-1273 arm at day 180, related to Figure 4.

	**mRNA-1273 (n=10)**															
	**G614**		**Delta**		**BA.1**		**BA.5**		**BQ.1.1**		**XBB.1.5**		**BA.2.86**		**JN.1**	
	**n**	**%**	**n**	**%**	**n**	**%**	**n**	**%**	**n**	**%**	**n**	**%**	**n**	**%**	**n**	**%**
**<100, n (%)**	0	0.0	0	0.0	1	10.0	1	10.0	2	20.0	5	50.0	2	20.0	4	40.0
**>100 to <300, n (%)**	1	10.0	1	10.0	1	10.0	1	10.0	4	40.0	5	50.0	5	50.0	3	30.0
**>300 to <1000, n (%)**	2	20.0	3	30.0	4	40.0	3	30.0	4	40.0	0	0.0	3	30.0	3	30.0
**≥1000, n (%)**	7	70.0	6	60.0	4	40.0	5	50.0	0	0.0	0	0.0	0	0.0	0	0.0

Table S15. Neutralizing response by NT_{50} categories against different SARS-CoV-2 variants in N+ subjects of BNT162b2 arm at day 180, related to Figure 4.

	BNT162b2 (n=18)															
	G614		Delta		BA.1		BA.5		BQ.1.1		XBB.1.5		BA.2.86		JN.1	
	n	%	n	%	n	%	n	%	n	%	n	%	n	%	n	%
**<100, n (%)**	0	0.0	0	0.0	0	0.0	1	5.6	2	11.1	5	27.8	2	11.1	8	44.4
**>100 to <300, n (%)**	0	0.0	0	0.0	2	11.1	0	0.0	8	44.4	8	44.4	9	50.0	6	33.3
**>300 to <1000, n (%)**	3	16.7	2	11.1	3	16.7	3	16.7	6	33.3	5	27.8	5	27.8	4	22.2
**≥1000, n (%)**	15	83.3	16	88.9	13	72.2	14	77.8	2	11.1	0	0.0	2	11.1	0	0.0

Table S16. Neutralizing response by NT_{50} categories against different SARS-CoV-2 variants in N+ subjects of mRNA-1273 arm at day 180, related to Figure 4.

	mRNA-1273 (n=11)															
	G614		Delta		BA.1		BA.5		BQ.1.1		XBB.1.5		BA.2.86		JN.1	
	n	%	n	%	n	%	n	%	n	%	n	%	n	%	n	%
**<100, n (%)**	0	0.0	0	0.0	0	0.0	0	0.0	3	27.3	5	45.5	2	18.2	3	27.3
**>100 to <300, n (%)**	0	0.0	0	0.0	0	0.0	2	18.2	4	36.4	4	36.4	4	36.4	7	63.6
**>300 to <1000, n (%)**	4	36.4	3	27.3	4	36.4	2	18.2	1	9.1	1	9.1	4	36.4	0	0.0
**≥1000, n (%)**	7	63.6	8	72.7	7	63.6	7	63.6	3	27.3	1	9.1	1	9.1	1	9.1
